# Application of Muscle Synergies for Gait Rehabilitation After Stroke: Implications for Future Research

**DOI:** 10.3390/neurolint16060108

**Published:** 2024-11-13

**Authors:** Jaehyuk Lee, Kimyung Kim, Youngchae Cho, Hyeongdong Kim

**Affiliations:** 1 Smart Technology Laboratory, Kongju National University, Cheonan-si 31080, Republic of Korea; jhl7589@kongju.ac.kr; 2 Department of Physical Therapy, School of Health and Environmental Science, College of Health Science, Korea University, Seoul 02841, Republic of Korea; bus238@naver.com (K.K.); yccho21c@hotmail.com (Y.C.)

**Keywords:** gait, muscle synergy, motor module, rehabilitation, stroke

## Abstract

Background/Objective: Muscle synergy analysis based on machine learning has significantly advanced our understanding of the mechanisms underlying the central nervous system motor control of gait and has identified abnormal gait synergies in stroke patients through various analytical approaches. However, discrepancies in experimental conditions and computational methods have limited the clinical application of these findings. This review seeks to integrate the results of existing studies on the features of muscle synergies in stroke-related gait abnormalities and provide clinical and research insights into gait rehabilitation. Methods: A systematic search of Web of Science, PubMed, and Scopus was conducted, yielding 10 full-text articles for inclusion. Results: By comprehensively reviewing the consistencies and differences in the study outcomes, we emphasize the need to segment the gait cycle into specific phases (e.g., weight acceptance, push-off, foot clearance, and leg deceleration) during the treatment process of gait rehabilitation and to develop rehabilitation protocols aimed at restoring normal synergy patterns in each gait phase and fractionating reduced synergies. Conclusions: Future research should focus on validating these protocols to improve clinical outcomes and introducing indicators to assess abnormalities in the temporal features of muscle synergies.

## 1. Introduction

According to the 2021 mortality statistics in South Korea, stroke is classified as a cerebrovascular disease and ranks as the fourth leading cause of death, with 44.0 deaths per 100,000 in the population in 2021 [1]. As a single disease, it is the second leading cause of death domestically. Despite the completion of standard rehabilitation processes, 50–60% of stroke survivors still experience motor impairments, and at least 50% of patients report partial limitations in their activities of daily living [2,3,4,5]. Therefore, the recovery of gait function during the rehabilitation phase after a stroke is one of the most crucial goals of treatment, significantly influencing prognosis, quality of life, and the patient’s return to society [6,7,8,9]. Over the past few decades, extensive research has been conducted on stroke gait rehabilitation, leading to remarkable advancements in gait rehabilitation methods based on quantitative and objective biomechanical analyses [10,11,12,13,14].

Gait is a daily movement that inevitably requires the central nervous system (CNS) to efficiently manage the myriad possibilities arising from redundant degrees of freedom in the musculoskeletal system [15,16,17,18]. According to numerous studies, patients with stroke exhibit pathological muscle activation patterns that deviate from normal gait patterns, such as hemiparesis, changes in muscle tone, and foot drop [19,20,21,22,23]. These deviations affect muscle synergies (MSs), defined as the coordinated activations of muscle groups to perform movement, which are increasingly being addressed in clinical studies [24,25,26,27,28]. MS analysis is used to investigate how the CNS regulates coordinated activation and has been consistently applied in gait analysis [29,30,31,32,33,34,35,36]. In patients with abnormal gait patterns, such as those with stroke, MSs are expressed differently compared to healthy individuals, ultimately leading to inefficient movement [24,37,38,39,40,41,42,43,44,45,46]. Many previous studies have reported abnormal MSs manifesting during gait in patients with stroke [46,47,48]. Recently, these findings have been applied to the development of electrical stimulation therapies, robotic prosthetics, and rehabilitation devices. Although a complete understanding of the neural mechanisms underlying gait remains unclear, MS analysis contributes to the fundamental understanding of abnormal gait through electromyography (EMG), which is widely used across medical centers because of its measurement practicality and time efficiency.

MS analysis addresses the degrees of freedom for problems occurring in neural and multi-joint musculoskeletal systems by distinguishing between spatial and temporal features through matrix decomposition and unsupervised machine learning algorithms [49,50,51,52]. The spatial features refer to the weight coefficients of each muscle in the synergies. These coefficients represent how strongly each muscle contributes to a particular MS. The spatial features of MS capture the distribution and contribution of different muscles to the overall movement pattern. Temporal features pertain to the activation profile of MSs, which describes how MSs experience changes over time during movement.

Various techniques, such as principal component analysis (PCA), independent component analysis (ICA), factor analysis (FA), mixed ICA/PCA, non-negative matrix factorization (NNMF), and autoencoders, have been used to analyze MS [53,54,55,56,57,58,59]. Owing to the use of various analytical techniques, understanding each method is essential for understanding consistency across studies, and this can simultaneously act as a barrier to effectively communicating research findings to clinicians. In addition, the findings vary across studies owing to the experimental conditions, synergy extraction criteria, and the number of muscles measured. Overall, most studies have consistently reported that stroke patients exhibit fewer MSs than healthy controls (e.g., healthy individuals typically exhibit three–five synergies, whereas stroke patients show three or fewer synergies) [24,40], although few studies have found no differences [41,44]. The reduction in MSs observed in stroke patients is often interpreted as a pathological phenomenon due to synergy merging, which leads to simplified motor control [60,61]. While many studies have primarily focused on MS during straight walking in patients with stroke, a recent finding reported that both the temporal and spatial features of MS during curved walking differ significantly from those in healthy controls, depending on the walking direction [42].

The coexistence of consistency and diversity in previous findings, along with variations in terminology among researchers (e.g., some studies refer to MSs as motor modules), highlight the need for a comprehensive view that could assist clinicians in designing rehabilitation strategies while also providing researchers with insights into the direction of future studies. Therefore, this study aimed to present state-of-the-art rehabilitation by providing a comprehensive review of MS using a systematic methodology to address motor control issues across each phase of gait.

## 2. Materials and Methods

Although this review adopted a comprehensive approach to explore the current literature on gait MS analysis and rehabilitation strategies in stroke patients, we followed a structured methodology inspired by the PRISMA guidelines to enhance the transparency and reproducibility of the review process [62,63]. By employing this hybrid methodology, we adhered to a structured framework that enhanced clarity and objectivity in the reporting of findings. It is important to note that while this study focused on the clinical interpretation of research outcomes rather than a qualitative assessment of each study’s quality, the methodology employed aimed to ensure a thorough and systematic review. This approach maximizes the benefits of the systematic review format and provides clear and consistent information to readers.

### 2.1. Search Strategy

A literature search was conducted using electronic databases such as PubMed, Scopus, and Web of Science, with publication dates ranging from January 2010 to May 2024. The search was performed using a query combining the following keywords, as agreed upon by the authors of this study: the disease of interest (“Stroke”); the area of rehabilitation interest (“Gait” OR “Walk”); and the research methods of interest (“muscle synerg*” OR “motor module*”). The search query was constructed as (“Stroke”) AND (“Gait” OR “Walk”) AND (“muscle synerg*” OR “motor module*”).

### 2.2. Study Eligibility Criteria

According to the search strategy, full-text journal articles related to MSs in ischemic stroke patients, including the keywords in the title and abstract, were reviewed. To comprehensively interpret and discuss the results based on the credibility and objectivity of experimental studies, the following types of papers were excluded from the review: (1) duplicate papers; (2) conference proceedings, book chapters, reports, letters, and review papers; (3) papers without Journal Citation Reports (Impact Factor, IF); (4) papers not written in English; (5) papers without gait task; and (6) papers without muscle synergy extraction. Finally, full-text papers that made it difficult to directly compare the pathological synergy between stroke patients and healthy controls were excluded.

### 2.3. Extraction of Study Characteristics

In the interpretation of MS research, it is essential to consider demographic information such as the characteristics of the participants (e.g., age), the measured muscles, the synergy extraction algorithms (e.g., NNMF), criteria (e.g., the variance accounted for to determine the minimum number of synergies), experimental gait tasks (e.g., overground walking), and the methods used for EMG signal processing (i.e., types of filters).

### 2.4. Integration of MS Features in Stroke Patients Compared to Controls

Despite the differences in experimental conditions and analytical techniques for synergy extraction across studies and the resulting variability in gait synergy patterns among stroke patients, this study aimed to integrate and compare synergy patterns in stroke patients. For temporal features, there was no visual methodology to integrate the results across studies. However, for spatial features, we visually compared the dominance of muscle weights within the synergies activated during the four gait phases (weight acceptance, push-off, foot clearance, and leg deceleration) and summarized these comparisons in a table for a clear presentation.

### 2.5. Extraction of Implications for Future Research

The four authors of this study conducted a cross-review to ensure that the results were not overinterpreted by deriving implications that reflected the interpretations of the researchers of each paper as accurately as possible. If a study was limited to reporting phenomena without providing detailed interpretations, the results were discussed until a consensus was reached among the authors. For the intervention studies, only the results of MS in stroke patients before the intervention were included in the review.

## 3. Results

### 3.1. Selected Studies

**Figure 1:**
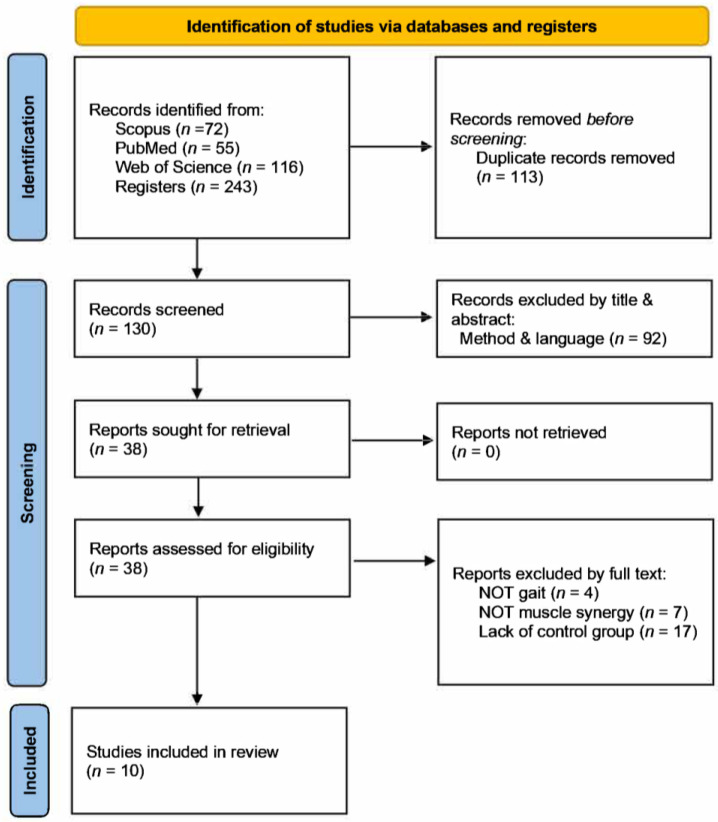
PRISMA flowchart for study inclusion/exclusion.

The final search strategy retrieved 243 studies from the three databases. A total of 113 duplicates were identified, leaving 130 studies for screening. No additional studies were found through manual reference list screening. The studies were first screened based on the title and abstract, excluding different parameters such as population, intervention, and outcomes. A total of 38 full texts were included for eligibility screening. Then, after reading the remaining 38 full texts, studies that met the exclusion criteria were removed. Eventually, 10 studies met all the inclusion and exclusion criteria and were included. Figure 1 shows a flowchart with a more detailed overview.

### 3.2. Study Characteristics

Most studies on MS in stroke patients’ gait have focused on chronic patients more than 6 months post-stroke in Table 1 (8 of 10 studies) [24,38,40,41,42,44,45,46]. The gait task was predominantly conducted using overground walking (7 of 10 studies) [39,40,41,42,43,44,45], and the most commonly employed MS extraction technique was NNMF (8 of 10 studies) [24,39,40,41,43,44,45,46]. In terms of extraction criteria, the total variance accounted for (VAF) was 90% or higher in seven out of ten studies [24,39,40,41,44,45,46]. Most signal filtering methods initially removed artifacts using a band-pass filter (20–750 Hz), followed by rectification and then a low-pass filter (4–40 Hz) to create the final linear envelope before extraction [24,39,40,41,42,43,44,45,46].

### 3.3. Number of MS in the Paretic Side of Limb by Stroke

For this analysis, two studies were excluded because the authors predetermined the number of MSs before extraction, which made it difficult to examine pathological changes [42,43]. The findings of the eight remaining studies are summarized in Table 2. Five identified a pathological reduction in MSs, which is referred to as the merging phenomenon [24,39,40,45,46].

### 3.4. MS Features in the Paretic Side of Limb by Stroke

Table 3 presents a comparison of the spatial features extracted from the MS analysis. A visual comparison of the spatial features of MS was possible in 10 studies; however, differences in temporal features were difficult to compare clearly. In terms of spatial features, the altered synergies in stroke patients showed significant changes in the rectus femoris (RF) during the weight acceptance phase [24,38,39,40,43,44,46], the tibialis anterior (TA) during the foot clearance phase [40,41,42,43,46], and hamstrings during the leg deceleration phase [38,40,41,42,45,46]. The calf muscles, which play key roles in the push-off phase, showed little or no change.

## 4. Discussion

Stroke recovery is predominantly achieved within the first three months after stroke onset, and the effectiveness of stroke rehabilitation gradually diminishes over time [64,65,66]. Therefore, specialized rehabilitation plays a crucial role during the acute phase of stroke [67]. Gait operates via a complex mechanism involving the integration of various sensory stimuli and motor signals from the CNS. However, considering that stroke patients often face difficulties in generating motor signals due to brain cell damage, the key is to maximize the sensory stimulation necessary for gait to promote the spontaneous recovery of brain cells and induce reversibility.

From this perspective, although MS analysis based on computational algorithms can provide valuable clinical insights into the mechanisms of motor control, there are limitations in applying these findings clinically because of the complexity of extraction principles and variability in results based on research methodologies. Therefore, this study aimed to enhance the clinical practicality and accessibility of MS in stroke gait rehabilitation and to suggest future research directions through a focused analysis and discussion of previous findings. Prior to conducting this review, we categorized the primary MSs based on a synthesis of studies as follows: synergy 1 (S1) for weight acceptance, synergy 2 (S2) for push-off, synergy 3 (S3) for foot clearance, and synergy 4 (S4) for leg deceleration.

### 4.1. Number of MSs in Stroke Gait

Most MS studies compared the number of synergies because they provide a straightforward quantitative measure of motor control complexity. Although there is variability in the reported number of synergies owing to the study’s methodology, such as the measured muscles, gait task, extraction algorithms, and criteria, the reduction in the number of synergies observed in stroke patients is interpreted as a synergy-merging phenomenon [24,39,40,45,46,68]. A decrease in the number of MSs is a prominent indicator of impaired motor control in stroke patients and is observed to be restored when these synergies are fractionated [69]. It has been reported that this reduction is significantly correlated with spatiotemporal gait parameters, such as walking speed, step length, and step width. [25,70]. In addition to the number of synergies, another critical metric is the VAF by a single synergy (VAF1), which represents the proportion of data variance explained by individual synergy. In patients with stroke, a higher VAF1 value is typically associated with fewer synergies, indicating the reduced ability of an individual synergy to explain the total data variance in muscle activities compared with healthy individuals [24,39,40,45,46]. Furthermore, some studies have reported differences in the number of synergies between paretic and non-paretic limbs [24,39,46]. Therefore, these results suggest that clinicians should provide task-oriented training for gait rehabilitation by dividing the gait cycle into stages and encouraging the fractionated use of key muscles at each stage. Furthermore, such training needs to be applied independently not only to the paretic limb but also to the non-paretic limb because some studies have reported that the number of synergies in the non-paretic limb is adjusted over time to match the decreased motor control abilities of the paretic limb through an adaptive mechanism. Thus, it would be appropriate to prevent the motor control simplification of the non-paretic limb during early rehabilitation while simultaneously setting a goal to restore the complexity of the simplified motor control in the paretic limb using task-oriented approaches [71,72]. Rehabilitation training is currently feasible because of the development of specialized gait rehabilitation robots and modality equipment that were not available in the past [73,74]. For example, one study reported that when the MS analysis results were applied to electrical stimulation therapy for gait rehabilitation, gait synergies were successfully restored [43].

### 4.2. Spatial Features of MS in Stroke Gait

This study is the first attempt to synthesize previous findings related to the spatial features of MS in patients with stroke. In doing so, we intended to facilitate a deeper understanding of the pathological patterns of muscle weakness in patients with stroke. As a result of comparing reports on spatial features, the key muscles that functioned differently from the controls were the RF, hamstring (semitendinosus [ST], biceps fermoris [BF]), and TA. For instance, the RF normally functions in S1 and S3; however, in stroke patients, it was observed to function in other synergies, such as S2 and S4, or to be excessively activated in a particular synergy [24,38,39,40,43,44,46]. In recent years, task-oriented rehabilitation has been widely adopted in stroke rehabilitation [75,76,77]. It has been shown to aid in the recovery of gait parameters as well as muscle synergy restoration [78,79]. However, task-oriented rehabilitation typically focuses on recovering overall movement patterns rather than on the recovery of individual synergies. Most stroke patients, however, experience pronounced impairments in specific muscle synergies rather than across all synergies. Therefore, it is necessary to target the recovery of individual synergies by specifically rehabilitating the muscles primarily involved in each synergy and restoring the relationships between muscles within a single synergy. This suggests that clinicians should provide rehabilitation protocols that focus not only on the individual strengthening of the RF, ST, BF, and TA muscles but also on activating the muscle groups that work within the same synergy during the corresponding gait phase in which issues arise. For instance, rehabilitation therapists should consider methods such as inducing simultaneous muscle contractions through palpation or using the patient’s posture and positioning to facilitate co-contraction.

### 4.3. Temporal Features of MS in Stroke Gait

In synergy research, temporal features have been relatively less emphasized than spatial features, which present challenges in comparing stroke patients and controls. However, temporal features should also be considered as major factors influencing gait performance [80]. In contrast to spatial features, consistently reported changes in temporal features across studies primarily occurred in S2, which plays a crucial role in propulsion during walking and is closely related to walking speed [41,42,43,44]. It is important to note that the temporal features of synergies, particularly when activation begins and reaches its peak, significantly affect the patient’s gait ability. An excessively early onset of specific synergies has been reported in patients with stroke, which causes the merging of spatial features and prevents synergies from fully performing their required functions during the gait cycle [24,40,42,45,46]. Specifically, our experimental results showed that when stroke patients performed curved walking, the activation peak timing of the temporal features varied with direction [42]. For curved walking with the paretic leg on the inside (ICW), there was a delay in the activation timing of S1 and S4, whereas for curved walking with the paretic leg on the outside (OCW), there was a significant delay in the activation timing of S1 and S2. Additionally, other studies have indicated that temporal features that should be clearly expressed sometimes manifest ambiguously, showing lower similarity to healthy individuals [41,43,45]. For example, a study that divided stroke patients into a high-functioning group (HFG) and a low-functioning group (LFG) and compared their MSs with those of healthy controls found that, based on spatial features alone, the LFG did not exhibit abnormal patterns compared with the HFG or healthy controls. However, temporal features revealed that the activation phase was not clearly expressed in the LFG. To emphasize, it is crucial that future research provides various indicators to quantify the regularity and clarity of the phase in temporal features.

### 4.4. Future Challenges for Gait Rehabilitation for Stroke Patients Based on MS

Several studies have found that the recovery of MSs is closely related to improvements in gait performance [40,44,81]. However, few studies have reported significant changes in the number of gait synergies after rehabilitation [82,83,84]. This suggests that while gait function indicators, such as speed and stride length, may improve through the recovery of muscle strength, muscle tone, and compensatory movements, the actual recovery of motor control may remain limited. This highlights the need for the ongoing development of rehabilitation strategies to address these limitations.

Another important consideration identified in previous studies is the substantial variability in MSs among patients. As the advancement of rehabilitation therapy must be tailored to the individual functionalities of patients, goal-oriented, and cost-effective, there is an urgent need for future research to explore how to rapidly enhance MS recovery in the early stages of stroke when recovery potential is at its highest, beyond the use of MS alone as a biomarker. Additionally, the development of standardized protocols for muscle synergy analysis seems necessary to improve comparability among studies in future research. This research contributes to the development and validation of diverse rehabilitation strategies aimed at recovering specific synergies.

Our study has several limitations. First, we did not review papers written in languages other than English. This limits our ability to analyze valuable information from a diverse range of researchers. Future research that includes papers in all languages, including English, would likely result in a more comprehensive review. Second, this study focused exclusively on ischemic stroke patients. We made this choice to eliminate inconsistencies in results that may arise from the differing gait patterns of ischemic and hemorrhagic stroke patients. Future research should consider including studies that focus on hemorrhagic stroke patients to provide a more comprehensive understanding.

In summary, based on the findings of the MS studies discussed above, future researchers and clinicians in the field should address the following key questions across three major aspects:

1. Features of MS: for the key muscles (i.e., RF, hamstring, and TA), which are changed primarily in spatial features, how can we restore the weighted activation within specific MSs, and how can we improve the recovery of the activation timing of S2?
2. Synergy merging and fractionation: How can we rapidly achieve the unmerging of MSs and enhance motor control complexity? Could single synergy-focused rehabilitation bring about significant changes not only in the recovery of muscle synergies but also in the unmerging of MSs?
3. Gait difficulty considerations: what should be addressed in rehabilitation when considering gait conditions: straight versus curved walking or slow versus fast walking?

## 5. Conclusions

This study aimed to derive the clinical and research implications from 10 MS studies through a comprehensive review using a systematic methodology. In conclusion, we emphasize that effective gait rehabilitation requires addressing both the paretic and non-paretic limbs independently and utilizing task-oriented training to maximize motor control complexity. This involves breaking down the gait cycle into distinct stages and focusing on the specific needs of each stage to enhance the rehabilitation outcomes. Future studies should focus on quantifying the temporal features of MSs and on developing tailored rehabilitation strategies to enhance recovery, including individual strategies for strengthening and restoring each synergy.

## Figures and Tables

**Table 1:** Characteristics of studies selected for the review.

Reference	Participants	Time Since Stroke	Gait Task	Extraction Algorithm	Extraction Criteria	Signal Processing
Clark, D.J. et al. (2010) [24]	55 patients, 59.5 ± 11.7 y M 35, F 20 Rt. 21, Lt. 34	57.8 ± 64.8 months	Treadmill	NNMF	Total VAF: >90%	High-pass-filtered (40 Hz), demeaned, rectified, and low-pass (4 Hz) Butterworth filter
Coscia, M. et al. (2015) [38]	12 patients, 58.5 ± 16.4 years M 9, F 3 Rt. 5, Lt. 7	54.6 ± 56.2 months	Treadmill, Overground Walking	FA	The number of retained synergies was identified using the criterion of the eigenvalue > 1; 3 synergies were extracted	Rectified and low-pass-filtered (10 Hz) Butterworth filter
Ebihara, A. et al., (2024) [39]	1 patient 49 years M 1 Lt. 1	33 days	Overground Walking	NNMF	Total VAF: >90%	Rectified and low-pass (40 Hz) Butterworth filter
Ferrante, S. et al., (2016) [40]	2 patients 67, 64 years M 2 Rt. 1, Lt. 1	C1: 11 years C2: 9 months	Overground Walking	NNMF	Total VAF: >90%	Band-pass-filtered (40–400 Hz), rectified, and low-pass (5 Hz) Butterworth filter
Gizzi, L. et al. (2011) [41]	10 patients 45.9 ± 16.5 years M 7, F3 Rt. 8, Lt. 2	12.0 ± 4.73 months	Overground Walking	NNMF	Total VAF: >80%	Band-pass filtered (20–400 Hz), rectified and low-pass-filtered (10 Hz) Butterworth filter
Lee, J. (2024) [42]	13 patients 63.2 ± 8.3 years M 7, F 6 Rt. 9, Lt. 4	5.0 ± 0.8 months	Overground Walking	Autoencoder	Fixed synergy extraction at 4	Band-pass filter (20–750 Hz), a high-pass filter (35 Hz), rectified, and low-pass filter (5 Hz) Butterworth filter
Lim, J. et al. (2021) [43]	2 patients 62, 60 years M 2 Rt. 1, Lt. 1	C1: 10 months C2: 2 months	Overground Walking	NNMF	Fixed synergy extraction at 4	Band-pass filter (40–400 Hz), rectified, and low-pass filter (5 Hz) Butterworth filter
Routson, R.L. et al. (2013) [44]	22 patients 57.3 ± 13.2 M 15, F 7 Rt. 8, Lt. 14	19.0 ± 13.0 months	Treadmill	NNMF	Total VAF: higher than 90%	High-pass filter (40 Hz), demeaned, and low-pass filter (10 Hz) Butterworth filter
Young, D.R. et al., (2022) [45]	2 groups, 8 patients each 58.1 ± 7.95 years M 14, F 2 Rt. 8, Lt. 8	HFG: 64.9 ± 38.5 months LFG: 58.1 ± 55.3 months	Overground walking	NNMF	Total VAF: >90%	Rectified and demeaned band-pass filter (10–450 Hz) and low-pass filter (7 Hz) Butterworth
Zhu, F. et al. (2021) [46]	10 patients 59.3 ± 6.8 years M 8, F 2 Rt. 5, Lt. 5	44.7 ± 35.2 months	Treadmill	NNMF	Total VAF: >90%	Band-pass filter (20–250 Hz), rectified, and low-pass filter (4 Hz) Butterworth

Abbreviations: C, case; F, female; HFG, height-functioning group; LFG, low-functioning group; Lt., left; M, male; NNMF, non-negative matrix factorization; Rt., right; VAF, variance accounted for.

**Table 2:** The number of synergies cited from the findings reported in eight studies.

Reference	Numbers of Synergy in Stroke	Numbers of Synergy in Controls	Merging
Clark, D.J. et al. (2010) [24]	2.7 synergies for the paretic leg, and 3.5 synergies for the non-paretic leg	3.6 synergies for the healthy right leg, and 3.7 synergies for the healthy left leg	Identified
Coscia, M. et al. (2015) [38]	3 synergies for the paretic leg	3 synergies for the healthy leg	None
Ebihara, A. et al. (2024) [39]	2-3 synergies for the paretic leg	3 synergies for the non-paretic leg	Identified
Ferrante, S. et al. (2016) [40]	3 synergies for the paretic leg	4 synergies for the healthy dominant leg	Identified
Gizzi, L. et al. (2011) [41]	4 synergies for the paretic leg, and 4 for the non-paretic leg	4 synergies for the healthy left leg	None
Routson, R.L. et al. (2013) [44]	4 synergies for the bilateral legs	4 synergies for healthy bilateral legs	None
Young, D.R. et al. (2022) [45]	1.38 synergies for the bilateral legs	2 synergies for healthy bilateral legs	Identified
Zhu, F. et al. (2021) [46]	3.1 synergies for the paretic leg, and 3.8 synergies for the non-paretic leg	4.45 synergies for bilateral legs	Identified

**Table 3:** The comparison of spatial features of synergies in stroke compared to the controls.

Reference	Study Design	Classification	Involved Primary Muscles												
			**GM**	**Gm**	**TFL**	**RF**	**ADD**	**VM**	**ST**	**BF**	**LG**	**MG**	**SO**	**PEL**	**TA**
Clark, D.J. et al. (2010) [24]	Cross-sectional (vs healthy)	None	$S_{\;WA}^{WA}$			$S_{\;WA}^{WA,FC}$		$S_{\;WA}^{WA}$	$S_{LD}^{LD}$	$S_{LD}^{LD}$		$S_{PO}^{PO}$	$S_{PO}^{PO}$		$S_{FC}^{FC}$
Coscia, M. et al. (2015) [38]	Cross-sectional (vs healthy)	None	$S_{WA}^{WA}$	$S_{WA}^{WA}$	$S_{WA}^{WA}$	$S_{WA}^{WA,\;FC,LD}$	$S_{WA}^{FC}$	$S_{WA}^{WA}$	$S_{WA}^{WA,FC,LD}$	$S_{WA}^{WA,\;FC}$		$S_{PO}^{PO}$	$S_{PO}^{PO}$	$S_{PO}^{PO}$	$S_{FC}^{FC}$
Ebihara, A. et al., (2024) [39]	Case–control (vs non-paretic)	C1	$S_{WA}^{WA}$			$S_{WA}^{FC}$	$S_{WA}^{WA}$		$S_{LD}^{LD}$		$S_{PO}^{PO}$	$S_{PO}^{PO}$	$S_{PO}^{PO}$		$S_{FC}^{FC}$
Ferrante, S. et al., (2016) [40]	Case–control (vs healthy)	C1	$S_{WA,FC}^{WA}$			$S_{WA,LD}^{FC}$	-	$S_{LD}^{WA}$	$S_{LD}^{LD}$	$S_{LD}^{LD}$	-	$S_{PO}^{PO}$	$S_{PO}^{PO}$		$S_{WA,FC}^{FC}$
		C2	$S_{WA}^{WA}$			$S_{WA}^{FC}$		$S_{WA}^{WA}$	$S_{FC,LD}^{LD}$	$S_{FC,LD}^{LD}$		$S_{PO}^{PO}$	$S_{PO}^{PO}$		$S_{FC,LD}^{FC}$
Gizzi, L. et al., (2011) [41]	Cross-sectional (vs healthy)	None	$S_{\;WA}^{WA}$			$S_{\;FC}^{FC}$		$S_{\;FC}^{FC}$		$S_{\;FC}^{LD}$		$S_{PO}^{PO}$	$S_{PO}^{PO}$		$S_{LD}^{FC}$
Lee, J. (2024) [42]	Cross-sectional (vs healthy)	ICG	$S_{WA}^{WA}$			$S_{WA,FC}^{WA,FC}$	$S_{FC}^{FC}$	$S_{WA}^{WA}$	$S_{PO,\;FC,\;LD}^{LD}$	$S_{WA}^{WA,LD}$		$S_{PO}^{PO}$			$S_{FC}^{WA}$
		OCG	$S_{WA}^{WA}$			$S_{WA,FC}^{WA,FC}$	$S_{FC}^{FC}$	$S_{WA}^{WA}$	$S_{LD}^{LD}$	$S_{WA}^{LD}$		$S_{PO}^{PO}$			$S_{FC}^{WA}$
Lim, J. et al., (2021) [43]	Case–control (vs healthy)	C1	$S_{WA}^{WA}$			$S_{WA,\;FC}^{WA}$	$S_{FC}^{FC}$	$S_{WA}^{WA}$	$S_{LD}^{LD}$	$S_{LD}^{LD}$		$S_{PO}^{PO}$			$S_{FC,\;LD}^{WA,\;FC}$
		C2	$S_{WA}^{WA}$			$S_{PO}^{WA}$	$S_{FC}^{FC}$	$S_{PO}^{WA}$	$S_{LD}^{LD}$	$S_{LD}^{LD}$		$S_{PO}^{PO}$			$S_{PO,\;FC}^{WA,\;FC}$
Routson, R.L. et al. (2013) [44]	Cross-sectional (vs healthy)	None	$S_{\;WA}^{WA}$			$S_{\;WA}^{WA,\;FC}$		$S_{\;WA}^{WA}$	$S_{LD}^{LD}$	$S_{LD}^{LD}$		$S_{PO}^{PO}$	$S_{PO}^{PO}$		$S_{FC}^{FC}$
Young, D.R. et al., (2022) [45]	Cross-sectional (vs healthy)	HFG	$S_{\;WA,LD}^{WA,LD}$	$S_{\;WA,LD}^{WA,LD}$		$S_{\;WA,LD}^{WA,LD}$		$S_{\;WA,LD}^{WA,LD}$		$S_{\;WA,LD}^{WA,FC,\;LD}$		$S_{PO}^{PO}$	$S_{PO}^{PO}$		$S_{WA,\;FC,LD}^{WA,\;FC,LD}$
		LFG													
Zhu, F. et al., (2021) [46]	Cross-sectional (vs. non-paretic)	None	$S_{PO,\;FC,\;LD}^{WA,PO,LD}$			$S_{WA,PO}^{WA}$		$S_{WA,PO}^{WA}$	$S_{WA,\;LD}^{FC,\;LD}$	$S_{WA,\;LD}^{FC,\;LD}$		$S_{WA,\;PO}^{WA}$	$S_{WA,\;PO}^{WA}$		$S_{FC}^{WA,FC,\;LD}$

The areas shaded in light gray within the table represent the spatial features of the stroke patients in the study, which differed from those of the controls. S refers to the synergy, with the superscript indicating the gait phase in which the muscle prominently contributed to the synergy in controls and the gait phase in which the muscle contributed to the synergy in stroke patients. Abbreviations: ADD, adductor; BF, biceps fermoris; C, case; FC, foot clearance; GM, gluteus maximus; Gm, gluteus medius; HFG, height functioning group; ICG, inside curved walking; LD, leg deceleration; LFG, low-functioning group; LG, lateral gastrocnemius; MG, medial gastrocnemius; OCG, outside curved walking; PEL, peroneus longus; PO, push-off; RF, rectus femoris; SO, soleus; ST, semitendinosus; TA, tibialis anterior; TFL, tensor fasciae latae; VM, vastus medialis; WA, weight acceptance.

## Data Availability

Data can be made available on request by the authors.
